# Supplementary material for: Quantifying the Relationship between SARS-CoV-2 Wastewater Concentrations and Building-Level COVID-19 Prevalence at an Isolation Residence: A Passive Sampling Approach
Source: Int J Environ Res Public Health. 2022 Sep 7;19(18):11245. doi: 10.3390/ijerph191811245 (PMC9517461; doi:10.3390/ijerph191811245)
Supplement: Supplementary file 1 [file ijerph-19-11245-s001.zip › ijerph-1866259-supplementary.pdf]

## Supplemental Document

### Wastewater Concentration Calculations

The Cq value recorded by the PCR machine is first measured against the standard curve of known copies of SARS-CoV-2 genome. The signal is converted from Cq values to the concentration in the PCR using the constants generated by the standard curve for each plate: y-intercept, y_int, and slope (m)

Eqn. S1 $pcr_{conc} = e^{\frac{(CT-y\_int)}{m}}$ $\quad units = \frac{gene\ copies}{\mu L\ of\ PCR\ mixture}$

Divide by the volume of cDNA template in each well, multiply by the 20 $\mu L$ PCR volume

Eqn. S2 $\frac{gene\ copies}{\mu L\ PCR} \times \frac{20\ \mu L\ PCR}{vol.\ cDNA\ template} = \frac{gene\ copies}{\mu L\ \text{cDNA}}$

The RT reaction prior to PCR was assumed to be 100% efficient, meaning the concentration of cDNA in the reaction is equal to the initial RNA concentration

Eqn. S3 $RT_{eff} = \frac{conc\ cDNA}{conc\ RNA} = 1$

Convert to gene copies in the entire RNA stock by multiplying by the total volume of RNA extracted.

Eqn. S4 $\frac{gene\ copies}{\mu L\ \text{RNA}} \times V_{extracted} = gene\ copies$

RNA was extracted from ½ of a filter paper. Multiply by 2 to get the gene copies per whole filter.

Eqn. S5 $gene\ copies\ \times\ 2 =\ gene\ copies$

Divide by the volume of liquid phase sample filtered to obtain the concentration in the **eluted** sample solution.

Eqn. S6 $\frac{gene\ copies}{vol\ filtered} = gene\ copies\ /\ L$

Calculate the proportion of sample volume in the eluent, PSE. $V_{dilute}$ is the volume of water added to the sample to help elute particles from the tampon. $V_{elute}$ is the total volume obtained from the tampon sample, including $V_{dilute}$.

Eqn. S7 $PSE = \frac{V_{elute} - V_{dilute}}{V_{elute}}$ $\quad units = \frac{mL}{mL} = [\,]$

Multiply the result from Eqn. 7 to obtain the concentration in the **eluted** sample.

Eqn. S8 $\frac{gene\ copies}{L} \times PSE = \frac{gene\ copies}{L}$

Full calculations summarized:

Eqn. S9

$$PCR_{conc} \times \frac{20\ \mu L}{vol.\ cDNA\ template} \times V_{extracted} \times \frac{2}{V_{filtered}} \times PSE = \frac{gene\ copies}{L}\ of\ wastewater\ in\ eluted\ sample$$

**Supplemental Table S1.** Demographic and clinical characteristics of isolation building occupants

| Characteristic | Value |
|---|---|
| Sex | |
| Male (%) | 59.4 |
| Female (%) | 40.6 |
| Reported any symptoms (%) | 92.3 |

**Supplemental Table S2. Primer sequences used for RT-qPCR in this study**

| Target | Primer Name | Oligonucleotide Sequence (5’→3’) |
|---|---|---|
| N1 | 2019-nCov_N1-R | 5’-TCTGGTTACTGCCAGTTGAATCTG-3’ |
| | 2019-nCov_N1-F | 5’-GACCCCAAAATCAGCGAAAT-3’ |
| N2 | 2019-nCov_N2-R | 5’-GCGCGACATTCCGAAGAA-3’ |
| | 2019-nCov_N2-F | 5’-TTACAAACATTGGCCGCAAA-3’ |
| BRSV | BRSV-R | 5’-ACACTGTAATTGATGACCCCATTCT-3’ |
| | BRSV-F | 5’-GCAATGCTGCAGGACTAGGTATAAT-3’ |

**Model Evaluation**

To evaluate the negative binomial model, we calculated McFadden’s pseudo $R^2$ value as a goodness-of-fit measure, which was found to be 0.20 indicating that our model performs well (McFadden, 1977). This statistic was generated comparing the null model to the fitted model using the following equation:

$$R^2 = 1 - \frac{\log \hat{L}(M_{Full})}{\log \hat{L}(M_{Intercept})} \quad (1)$$

Where $\hat{L}$ represents the maximized likelihood value from both our fitted model ($M_{Full}$) and the null model ($M_{Intercept}$).

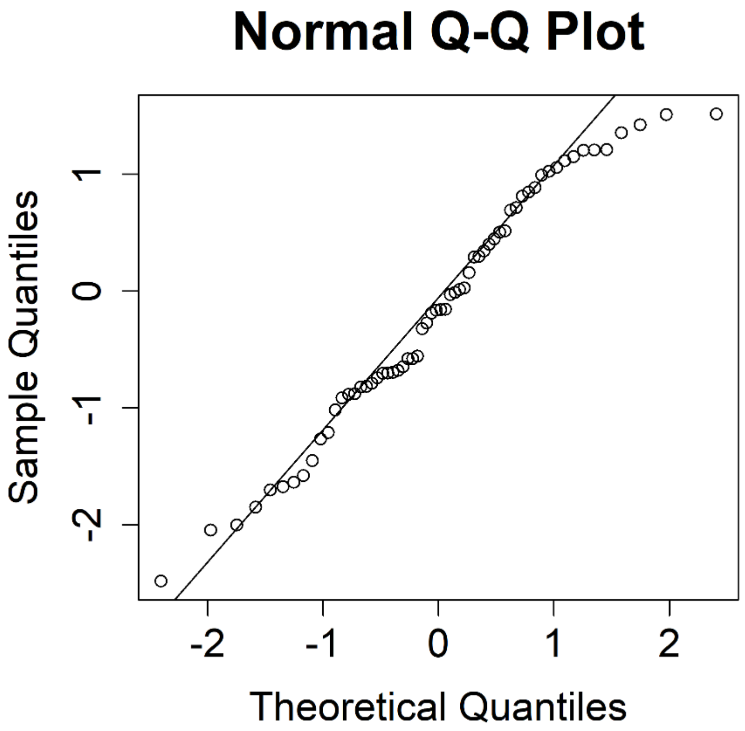


**Supplemental Figure S1:** Q-Q Plot to assess data distribution underlying our final negative binomial model

Reference

McFadden, D., 1977. Quantitative Methods for Analyzing Travel Behaviour of Individuals: Some Recent Developments. *Cowles Foundation for Research in Economics, Yale University*. Available at: https://ideas.repec.org/p/cwl/cwldpp/474.html.
